# In vitro characterization of PlyE146, a novel phage lysin that targets Gram-negative bacteria

**DOI:** 10.1371/journal.pone.0192507

**Published:** 2018-02-06

**Authors:** Yu Larpin, Frank Oechslin, Philippe Moreillon, Grégory Resch, José Manuel Entenza, Stefano Mancini

**Affiliations:** Department of Fundamental Microbiology, University of Lausanne, Lausanne, Switzerland; Universidade de Lisboa Faculdade de Medicina, PORTUGAL

## Abstract

The recent rise of multidrug-resistant Gram-negative bacteria represents a serious threat to public health and makes the search for novel effective alternatives to antibiotics a compelling need. Bacteriophage (Phage) lysins are enzymes that hydrolyze the cell wall of bacteria and represent a promising alternative to tackle this ever-increasing problem. Despite their use is believed to be restricted to Gram-positive bacteria, recent findings have shown that they can also be used against Gram-negative bacteria. By using a phage genome-based screening approach, we identified and characterized a novel lysin, PlyE146, encoded by an *Escherichia coli* prophage and with a predicted molecular mass of ca. 17 kDa. PlyE146 is composed of a C-terminal cationic peptide and a N-terminal N-acetylmuramidase domain. Histidine-tagged PlyE146 was overexpressed from a plasmid in *Lactococcus lactis* NZ9000 and purified by NI-NTA chromatography. PlyE146 exhibited *in vitro* optimal bactericidal activity against *E*. *coli* K12 (3.6 log_10_ CFU/mL decrease) after 2 h of incubation at 37°C at a concentration of 400 μg/mL in the absence of NaCl and at pH 6.0. Under these conditions, PlyE146 displayed antimicrobial activity towards several other *E*. *coli*, *Pseudomonas aeruginosa* (3 to 3.8-log_10_ CFU/mL decrease) and *Acinetobacter baumannii* (4.9 to >5-log_10_ CFU/mL decrease) strains. Therefore, PlyE146 represents a promising therapeutic agent against *E*. *coli*, *P*. *aeruginosa* and *A*. *baumannii* infections. However, further studies are required to improve the efficacy of PlyE146 under physiological conditions.

## Introduction

The massive use of antibiotics has led to a rise of bacteria that are multidrug-resistant (MDR) [1]. This phenomenon is particularly alarming in Gram-negative bacteria, including *Escherichia coli*, *Klebsiella pneumoniae*, *Pseudomonas aeruginosa* and *Acinetobacter baumannii*, which are responsible for a broad spectrum of infections that could become untreatable [2,3]. The rise of these MDR bacteria and the shortage of novel antibiotics warrants the search and the development of new alternative antibacterial agents [1].

Bacteriophage lysins, namely phage-encoded enzymes that hydrolyze the cell wall peptidoglycan of phage-infected bacteria for progeny release [4–6], represent a very promising novel alternative class of antimicrobial agents. In fact lysins, which generally have a broader spectrum activity than the producing phage, can ideally be exploited to eliminate specific bacterial infections [4]. Other advantages offered by lysins are the rapidity of action, high efficiency and the low chance to develop bacterial resistance since they target peptidoglycan components that are essential for bacterial viability [7–9]. A possible drawback of lysin therapy could be the generation of neutralizing antibodies, as they are exogenous proteins. However, several studies have shown that antibodies specific for lysins do not block their activity *in vitro*, and anaphylaxis was not observed in mice after repeated administration, implying that they can be utilized more than once [10].

The use of lysins as potential therapeutic agents has been widely investigated against Gram-positive bacteria, where when externally applied can induce rapid bacterial lysis due to the direct accessibility to the peptidoglycan [4,11–13]. By contrast, Gram-negative bacteria possess an outer membrane which protects the peptidoglycan from hydrolysis by phage lysins when applied from outside [14]. Consistent to this notion, only a few lysins with intrinsic antimicrobial activity against Gram-negative bacteria have been reported so far [15–19]. As an example, using a phage genome-based analysis Lai et al. have identified two lysins (A1S-1600 and A1S-2016) which were active against several *Acinetobacter* spp. [20]. Using the same approach, Lood et al. have more recently identified a lysin, namely PlyF307, which efficiently killed *A*. *baumannii* in vitro and in vivo [19]. Of remark, PlyF307 is composed of an N-terminal N-acetylmuramidase (lysozyme) domain [19] and a highly positively charged C-terminal domain, suggesting that penetration through the outer membrane and subsequent hydrolysis of the peptidoglycan substrate from the lysins may be mediated by cationic peptides. This concept was supported by the observation that just the cationic peptides retained substantial antimicrobial activity towards *A*. *baumannii* [21]. Consistent to this notion, recent studies have shown that lysins can be engineered to become active against Gram-negative bacteria by fusing the N- or C- terminus with cationic peptides [22–24]. However, reports of lysins active against Gram-negative bacteria remain limited and mostly focus on *A*. *baumannii* and *P*. *aeruginosa*.

In an attempt to identify novel lysins naturally active against *E*. *coli*, the C-terminal cationic peptide of PlyF307, alias P307, was used as a query to search for translated proteins sequences with similar properties in the *E*. *coli* genomes deposited in the NCBI database. In this study, a novel *E*. *coli* phage lysin, namely PlyE146, was identified and its antimicrobial activity against a panel of Gram-negative and Gram-positive bacteria was evaluated. PlyE146 was shown to efficiently kill in vitro several strains of *E*. *coli*, *P*. *aeruginosa* and *A*. *baumannii*.

## Materials and methods

### Bacterial strains and growth conditions

The bacterial strains used in this study are described in Table 1. They include a broad range of reference strains as well as clinical isolates. *E*. *coli*, *K*. *pneumoniae*, *P*. *aeruginosa*, *A*. *baumannii* and *Salmonella enterica* strains were grown at 37°C in LB medium (Difco Laboratories, Detroit, MI). *Staphylococcus aureus* was cultured at 37°C in tryptic soy broth (TSB; Difco) and *Streptococcus mitis* was grown at 37°C in brain-heart infusion (BHI; Difco). *L*. *lactis* NZ9000, used as a host for protein overexpression, was grown at 30°C in M17 medium (Difco) supplemented with 0.5% [wt/vol] glucose (GM17).

**Table 1:** Bacterial strains used in this study.

Strain	Source and Relevant characteristic(s)^a^
Gram-negative	
*E*. *coli* DH5α	Reference strain
*E*. *coli* K12	Reference strain
*E*. *coli* 8542	Strain from patient with sepsis, ESBL-producer
*E*. *coli* S115	Strain from patient with urinary tract infection, Imipenem-R,Colistin-R
*E*. *coli* 17	Strain from patient with sepsis, Amoxicillin-S, Ciprofloxacin-S
*E*. *coli* 23	Strain from patient with sepsis, Amoxicillin-R, Ciprofloxacin-R
*E*. *coli* 35	Strain from patient with peritonitis, Amoxicillin-S, Ciprofloxacin-S
*E*. *coli* 52	Strain from patient with skin infection, Amoxicillin-S, Ciprofloxacin-S
*K*. *pneumoniae* 8354	Strain from patient with sepsis, ESBL-producer
*S*. *enterica*	Strain from patient with bacteremia
*P*. *aeruginosa* ATCC 27853	Reference strain
*P*. *aeruginosa* UR1156	Strain from patient with bacteremia, Imipenem-R
*P*. *aeruginosa* 2062	Strain from patient with sepsis, Imipenem-R
*A*. *baumannii* H12555	Strain from burn patient, Ciprofloxacin-R
*A*. *baumannii* 33	Strain from patient with sepsis, Imipenem-R
*A*. *baumannii* FER	Strain from patient with sepsis, AmpC β-lactamase-producer, Imipenem-R
Gram-positive	
*S*. *aureus* ATCC 25913	Reference strain
*S*. *mitis* 859	Nasopharynx isolate, Penicillin-R
*L*. *lactis* NZ9000 pNZ8148	Expression strain, Chloramphenicol-R

^a^ESBL: extended spectrum β-lactamase; R: resistant

### Bioinformatics analysis

The peptide P307 (amino acids 108 to 138 of PlyF307) [19] was blasted against all the *E*. *coli* proteomes available in the protein database of NCBI. The top hit was one lysin (GenBank accession N° EKK47578) of 146 amino acids encoded from an *E*. *coli* 8.0569 prophage. The protein, that we named PlyE146, was found to possess a C-terminal domain with 68% sequence identity to P307.

### Cloning of lysin *plyE146*

*plyE146* was synthetized by Eurofins Genomics (Ebersberg, Germany) and cloned in the pET-28a expression vector (Novagen, Madison, WI) using NcoI and XhoI restriction sites. The resulting plasmid expressed PlyE146 fused to a 6His tag at its C-terminus was electroporated into electrocompetent *E*. *coli* BL21 (DE3). Isopropyl β-D-1-thiogalactopyranoside (IPTG)-mediated induction of *plyE146* expression led to growth arrest of logarithmic *E*. *coli* BL21 cells. Therefore, the *plyE146*-*6His* gene was sub-cloned into the *Lactococcus lactis* nisin-controlled gene expression system (NICE) pNZ8148, resulting in the construct pNZ8148-*plyE146-6His*. Briefly, *plyE146-6His* was amplified from the pET28a-plyE146 construct by polymerase chain reaction (PCR) using primers sml289 (5’-GAAATAATTTTGTTTAACTTTAAGAAGGAG-3’) (forward), which is complementary to a sequence upstream the NcoI recognition site and sml293 (5’-ATATATCTAGAGACCCGTTTAGAGGCCCCAAGG-3’) (reverse), containing the XbaI recognition site (underlined). The resulting DNA fragment of 613 bp was digested with NcoI and XbaI restriction enzymes (Promega, Madison, Wisconsin, USA) and ligated into pNZ8148, also digested with the same enzymes. The ligation mixture was electroporated into electrocompetent *L*. *lactis* NZ9000 cells. Transformants were selected on GM17 plates containing 10 μg/ml chloramphenicol. The presence of the insert into pNZ8148 was verified by commercial DNA sequencing (GATC Biotech, Konstanz, Germany).

### Protein overexpression and purification

PlyE146 was overexpressed from pNZ8148-*plyE146-6His* in *L*. *lactis* NZ9000 cells. Cells were grown aerobically in GM17 containing 10 μg/ml of chloramphenicol at 30°C with gently shaking (40 r.p.m.) until the mid-exponential phase (OD_600_ = 0.4). Protein expression was induced for 18 h at 30^o^ C by addition of 100 ng/ml of nisin.

Lactococcal cells containing overexpressed PlyE146 were harvested by centrifugation and then resuspended in 15 ml of binding buffer (100 mM NaH_2_PO_4_, 500 mM NaCl, 5 mM imidazole, pH 7.4). After incubation for 1 h at room temperature with 4 mg/ml of lysozyme, cells were disrupted by four passages through a French press at 30 MPa and incubated for 15 min on ice with 10 μg/ml RNAse and 10μg/ml DNase. The cell debris were removed by centrifugation (30 min at 8000 rpm). The cleared lysate (ca. 30 ml) was mixed with 2 mL of Ni-NTA resin (Qiagen, Hilden, Germany) and incubated overnight at 4°C in an end-to-end rotor. The slurry was then loaded onto a disposable polypropylene column and washed with 20 ml of binding buffer and 10 ml of washing buffer (100 mM NaH_2_PO_4_, 500 mM NaCl, 30 mM imidazole, pH 7.4). PlyE146-6His was eluted with 5 ml of elution buffer (100 mM NaH_2_PO_4_, 500 mM NaCl, 300 mM imidazole, pH 7.4). The eluate was dialyzed overnight against sodium phosphate monobasic dehydrate (NaPi) buffer (20 mM NaH_2_PO_4_•2H_2_O, pH 6.0). Purity of PlyE146 was determined by sodium dodecyl sulfate polyacrylamide gel electrophoresis (SDS-PAGE).

### Western blot analysis

Western blotting using an anti-histidine antibody was performed to confirm presence of the expected PlyE146 protein in the eluate. The protein samples were resolved by SDS-PAGE and then transferred to an Immobilon-P membrane (Millipore, Billerica, Massachusetts, USA) for 1.5 h at 60 V. After blocking with 5% (w/v) skimmed milk in PBS (milk/PBS), the membrane was incubated with 1:100 dilution of rabbit serum containing anti-6His antibody (final concentration, 1μg/mL) for 1 h. After four washes in milk/PBS, the membrane was incubated in milk/PBS with 0.5 μg/mL (final concentration) of a goat anti rabbit horseradish peroxidase (HRP) secondary antibody for 1 h. After four washes with PBS the blots were incubated with the western bright ECL solutions (Advansta, Menlo Park, CA) at a 1:1 ratio. The ECL signal was captured with a Fusion FX apparatus (Witec AG, Luzern, Switzerland).

### Antibacterial activity of PlyE146

The antibacterial activity of PlyE146 was initially tested against ca. 10^7^ CFU/mL logarithmic- and stationary-phase *E*. *coli* K12, which was used as reference strain. Bacterial cells were first washed in NaPi buffer (pH 6.0) and then incubated in the same buffer with different concentrations of PlyE146 (ranging from 3.1 to 500 μg/mL). Buffer without lysin was used as a negative control. After 2 h of incubation at 37°C with shaking (200 rpm) [19], samples were serially diluted and plated on agar plates. Viable cells were enumerated after overnight incubation of the plates at 37°C. Bactericidal concentration was defined as a ≥3 log_10_ decrease in cell counts as compared to that in the absence of lysin. Experiments were performed in triplicate and data expressed as mean ± standard deviation (SD).

To study the effect of pH and NaCl on the activity of PlyE146, logarithmic-phase *E*. *coli* K12 cells were incubated for 2 h at 37°C with 400 μg/mL of PlyE146 (the minimal bactericidal concentration,) in NaPi buffer with different pH values (6.0 to 9.0) or NaCl concentrations (0 to 500 mM).

Time-kill curve assays with exponentially growing (OD_600_ = 0.4) *E*. *coli* K12 cells (ca. 10^7^ colony forming units [CFUs]/mL) were performed under optimal conditions for PlyE146 bactericidal activity, namely in NaPi buffer (pH 6.0) with no NaCl and a protein concentration of 400 μg/mL After 0.5, 1, 2, 3 and 4 h of incubation at 37°C with shaking (200 rpm), aliquots were serially diluted and plated on agar plates. Viable cells were enumerated after overnight incubation of the plates at 37°C. Bactericidal concentration was defined as described above. Experiments were performed in triplicate and data expressed as mean ± SD.

To test the effect of the outer membrane-permeabilizer ethylenediaminetetraacetic acid (EDTA) on the activity of PlyE146, logarithmic- and stationary-phase *E*. *coli* K12 cells were pretreated with 5 mM EDTA before being incubated with PlyE146 (400 μg/mL) in NaPi buffer (pH 6.0) for 2 h at 37°C.

The spectrum of antimicrobial activity of PlyE146 (400 μg/mL) was tested against a panel of 16 Gram-negative bacteria (8 *E*. *coli*, 3 *P*. *aeruginosa*, 3 *A*. *baumannii*, 1 *K*. *pneumoniae* and 1 *S*. *enterica*) and 2 Gram-positive bacteria (1 *S*. *aureus* and 1 *S*. *mitis*). The assay was performed using bacteria in the exponential phase of growth using the aforementioned conditions. Viable cells were enumerated after 2 h of incubation. Experiments were performed in triplicate and data expressed as mean ± SD.

The effect of the serum on the activity of PlyE146 was analyzed with logarithmic growing *E*. *coli* K12, *P*. *aeruginosa* UR1156 and *A*. *baumannii* FER strains, three isolates that were efficiently killed in vitro (see Results) under optimal conditions for PlyE156 bactericidal activity (pH 6.0 and no salt). The experiments were conducted in triplicate in decomplemented human serum (by heating at 56°C for 30 min) and the results expressed as mean ± SD.

### Transmission electron microscopy (TEM)

TEM was used to visualize the effects of PlyE146 on bacterial cells. TEM was performed on mid-logarithmic phase (OD_600_ = 0.4) *A*. *baumannii* strain FER (used as a model organism) using 400 μg/mL of PlyE146 or buffer as control. After 1 h incubation, cells were fixed with glutaraldehyde at a final concentration of 25% and washed with PBS. Cells were then treated with metaperiodate (1% final concentration, 15 min incubation) and osmium tetroxide plus hexacyanoferate (1% and 1.5%, respectively. 1 h incubation). Cells were then centrifuged and the pellets were spun down in microcentrifuge tubes containing melted agar. After solidification of the agar, pellets were embedded in epon for ultra-thin sections (50 nm) preparation as described [25]. Micrographs were taken with a transmission electron microscope FEI CM100 (FEI, Eindhoven, the Netherlands) at an acceleration voltage of 80 kV with a TVIPS TemCam-F416 digital camera (TVIPS GmbH, Gauting, Germany).

## Results

### Identification of *plyE146* and purification of PlyE146

**Fig 1:**
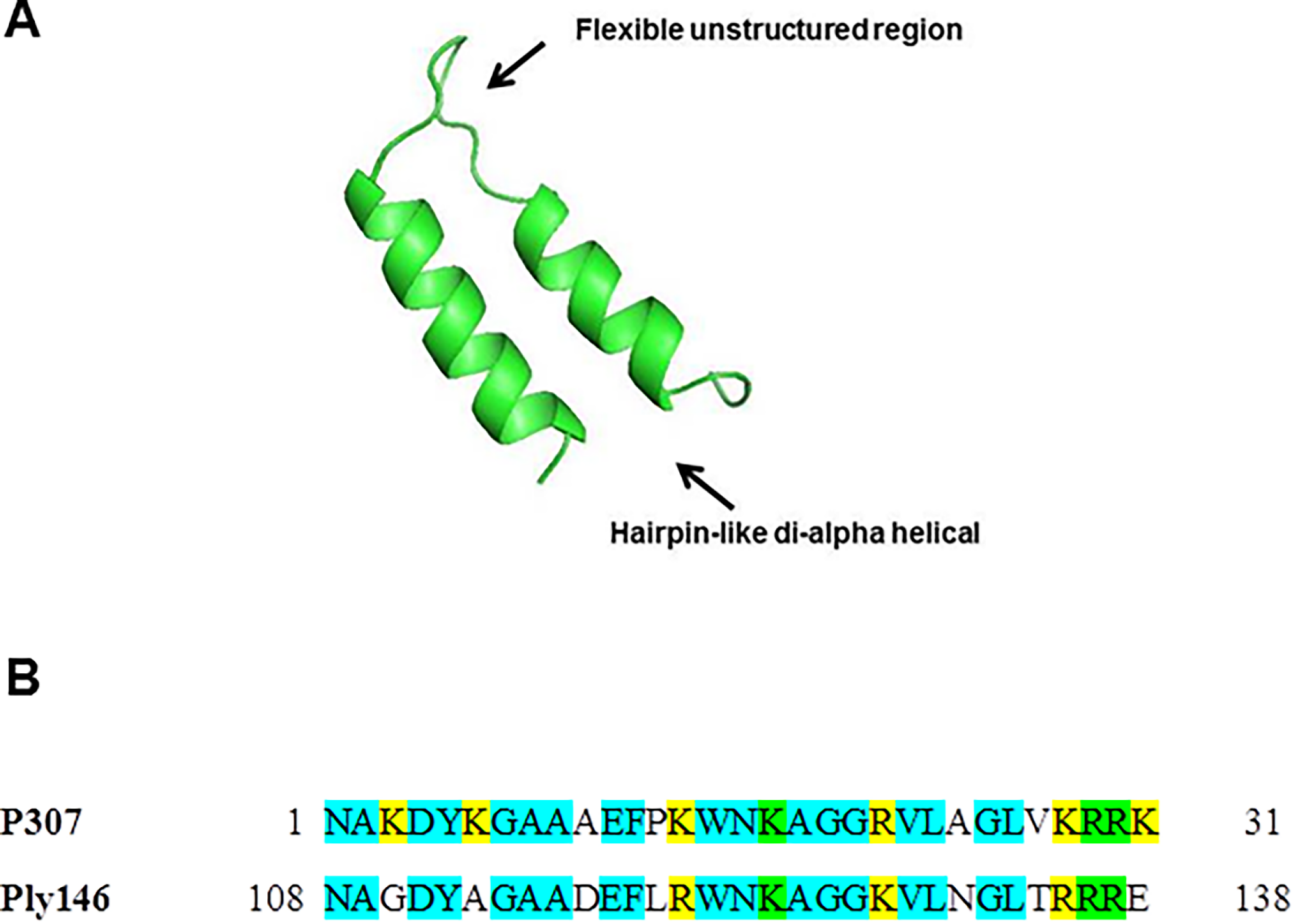
Structural prediction of the PlyE146 C-terminal domain and sequence alignment with P307. The C-terminal domain of PlyE146 (P146) assumes a hairpin-like di-alpha helical structure linked by a flexible unstructured region (A) and shares 68% identity with PF307 (B). In blue are conserved amino acids, in yellow positively-charged amino acids and in green conserved, positively-charged amino acids.

In an endeavor to identify natural lysins active against *E*. *coli*, the peptide P307 (amino acids 108 to 138 of the previously described lysin PlyF307 active against *A*. *baumannii*) [19] was used as a query against all the *E*. *coli* proteomes (translated protein sequences) available in NCBI. A putative bacteriophage lysin of 146 amino acids (GenBank accession N° EKK47578) encoded in the *E*. *coli* 8.0569 genome was the best hit. The putative lysin, that we named PlyE146, exhibits 57.7% sequence identity with PlyF307. The putative protein presents a N-terminal muraminidase domain and a C-terminal domain with a predicted hairpin-like di-alpha helical structure linked by a flexible unstructured region (Fig 1A; structure prediction generated using I-TASSER server [26,27]), which resembles that one of P307 (21). Moreover, the C-terminal peptide comprising amino acids 108–138, hereafter P146, exhibits 68% identity with P307 (Fig 1B). Notably, 6 of the 9 positively charged amino acid residues of P307 are also present in P146, despite only 3 are identical. In aggregate, the C-terminal domains of PlyE307 and PlyE146 exhibit a high degree of conservation.

To assess the antimicrobial activity of purified PlyE146, the encoding gene was cloned in the expression vector pET28a. *E*. *coli* cells were transformed with the resulting plasmid, expressing PlyE146 fused to a six-histidine tag (6His) at its C-terminus (pET28-PlyE146-6His). However, the expression of PlyE146-6His in exponentially growing *E*. *coli* cells led to growth arrest, suggesting a lethal effect exerted by PlyE146-6His. Thus, *plyE146-6His* was subcloned in an inducible expression system (pNZ8148) suitable for *L*. *lactis*. Overexpression in *L*. *lactis* NZ9000 and purification by Ni-NTA agarose affinity chromatography yielded a soluble protein (PlyE146-6His, from now on referred to as PlyE146) that migrated on SDS-PAGE at the expected molecular mass of 17 kDa.

### Western blot

To further verify the nature of the 17 kDa protein band, western blot was performed using primary rabbit anti-histidine antibodies and secondary goat anti-rabbit antibodies coupled to HRP. As expected, a chemiluminescent band with a molecular size of approximately 17 kDa was detected in the elution fraction, confirming its PlyE146-6His nature. Additional bands with higher molecular weights, possibly corresponding to protein oligomers, were also present in the eluate.

### PlyE146 antimicrobial activity

**Fig 2:**
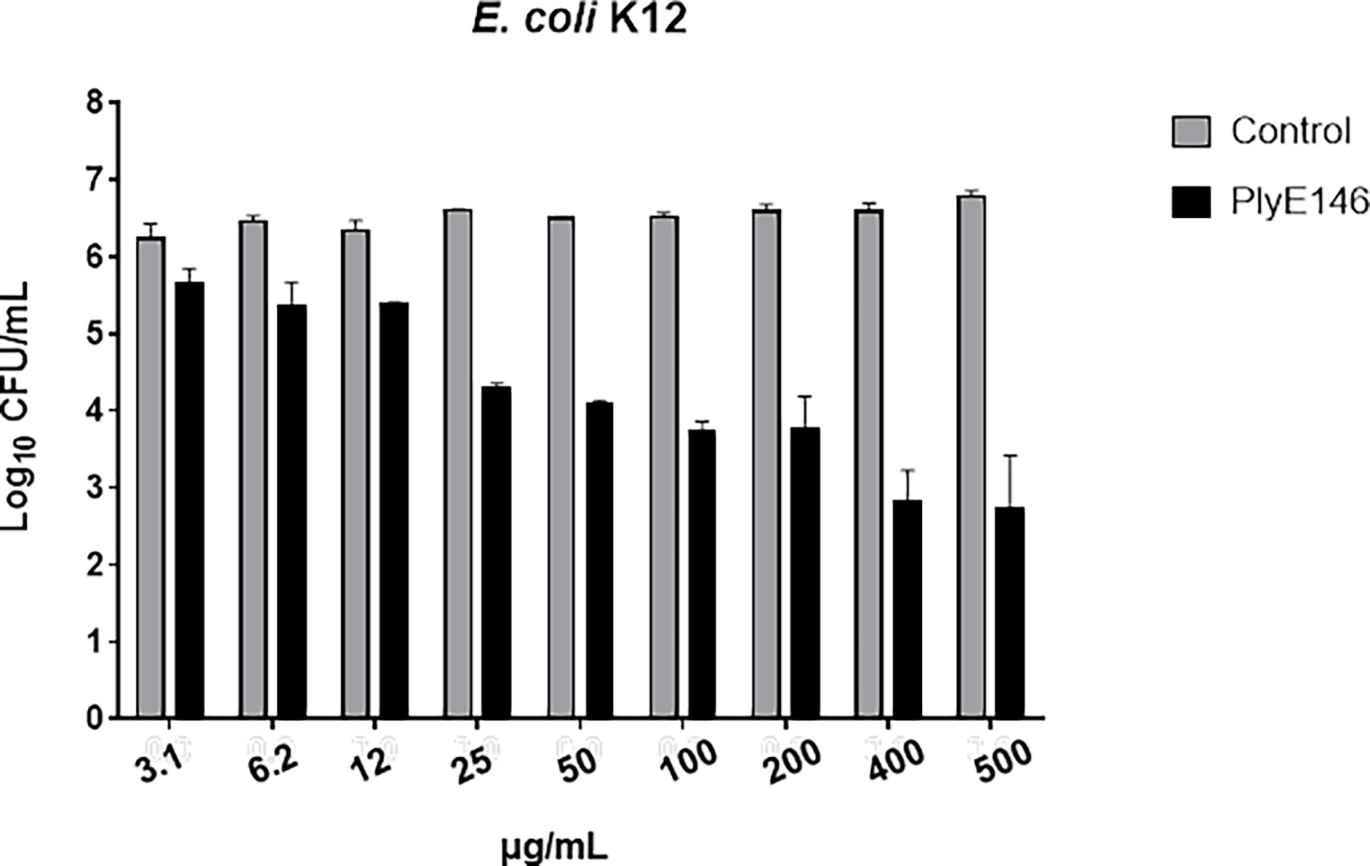
Bactericidal activity of PlyE146 on logarithmic growing *E*. *coli* K12 bacteria. *E*. *coli* K12 cells (10^6^ to 10^7^ CFU/mL) were exposed to increasing concentrations of PlyE146 in NaPi buffer (pH 6.0) at 37°C for 2 h, serially diluted and plated for colony counts. Results represent the mean ± standard deviation of triplicate experiments.

The bactericidal activity of PlyE146 against exponentially growing *E*. *coli* K12 cells was found to be concentration-dependent. In fact, while low PlyE146 levels (≤ 25 μg/mL) did not affect the viability of *E*. *coli* K12 after 2 h of incubation at 37°C, concentrations between 25 and 200 μg/mL reduced titers of *E*. *coli* K12 by ca. 2 log_10_ CFU/mL. The lowest concentration of PlyE146 with bactericidal activity (3.64 log_10_ kill) was 400 μg/mL (Fig 2). Similarly to other lysins [13,19,23], the antimicrobial activity of PlyE146 was significantly lower against stationary-phase cells (<1 log_10_ kill).

**Fig 3:**
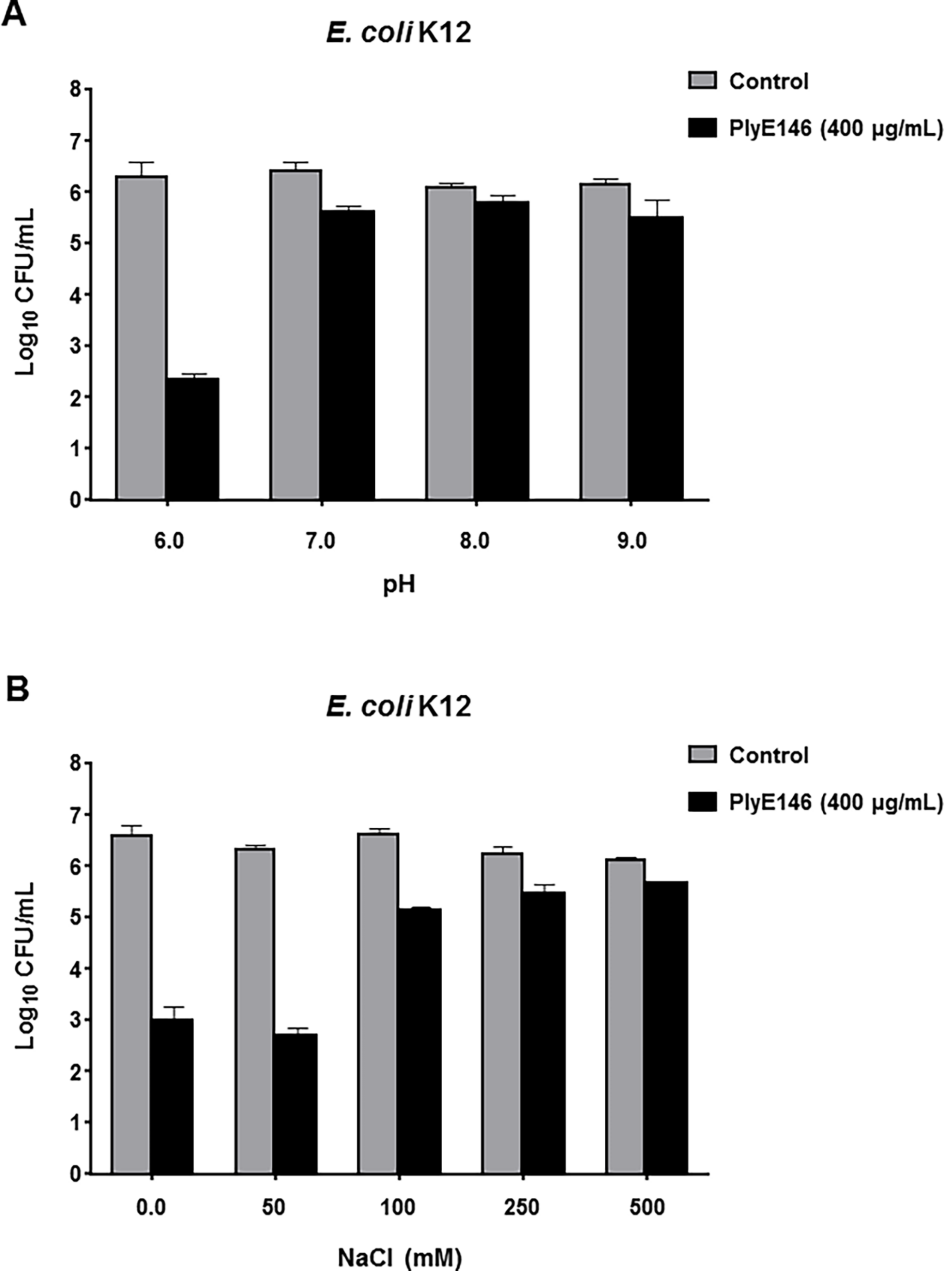
Effect of pH and NaCl on the antibacterial activity of PlyE146. To study the effect of pH (A) and NaCl (B) on *E*. *coli* K12, logarithmic growing cells were exposed to PlyE146 (400 μg/mL) in NaPi buffer at different pH and NaCl concentrations for 2 h at 37°C, serially diluted and plated for colony counts. Results represent the mean ± standard deviation of triplicate experiments.

The effects of pH and NaCl on the *in vitro* antibacterial activity of PlyE146 were also investigated. The highest activity of PlyE146 was observed at pH 6.0 (reduction of 4 log_10_ CFU/mL of the inoculum in buffer) (Fig 3). At higher pH, PlyE146 caused as little as ≤1 log_10_ reduction of cell viability (Fig 3). Moreover, NaCl exerted an inhibitory effect on PlyE146 activity. Indeed, while the activity of PlyE146 was essentially preserved in the presence of 50 mM NaCl, it significantly decreased in the presence of NaCl concentrations ≥100 mM (1.1 to 1.6 log_10_ CFU/mL reduction). Based on these results, we elected to continue the *in vitro* experiments by testing the bactericidal activity of PlyE146 after 2 h exposure against exponentially growing cells at pH 6.0 and at a concentration of 400 μg/mL.

**Fig 4:**
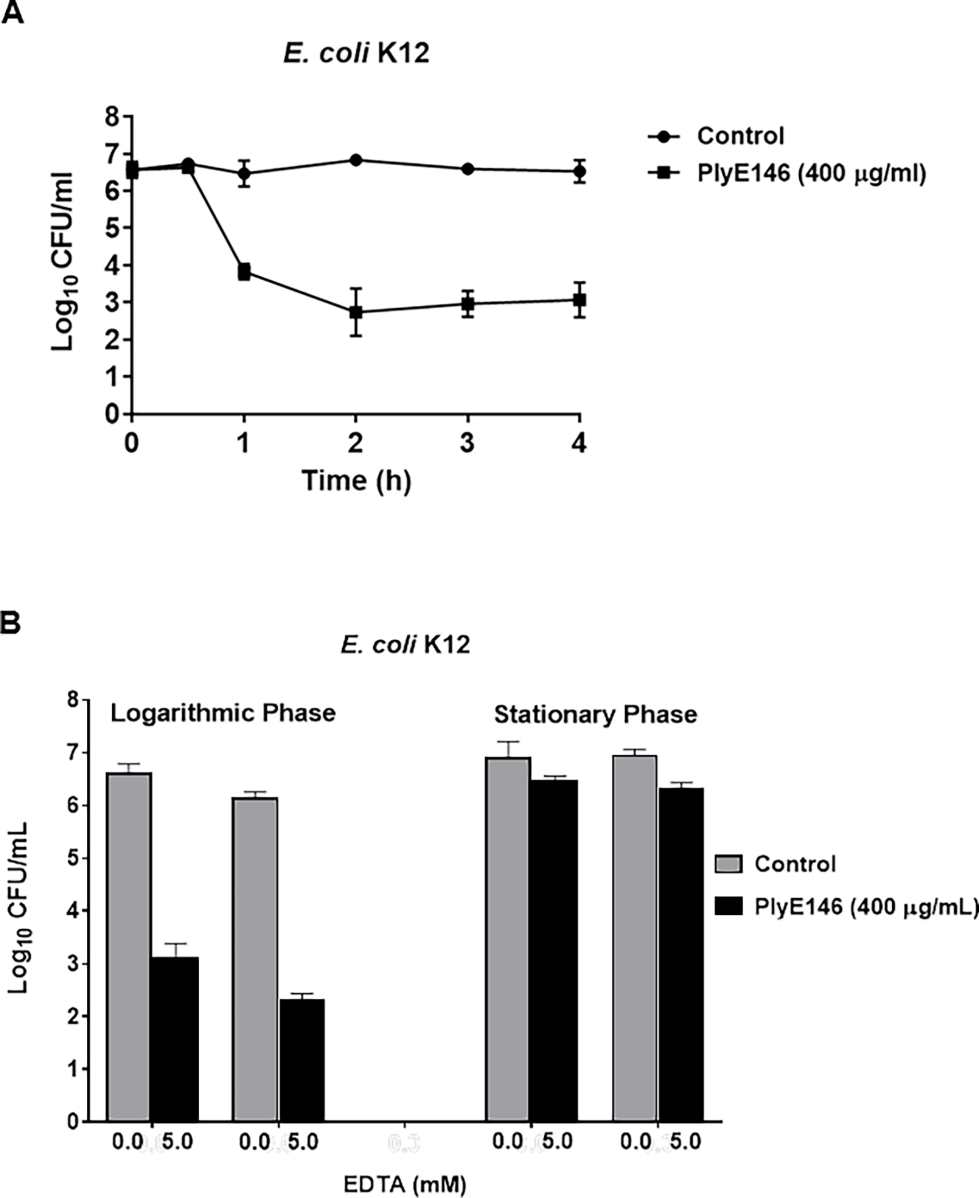
Time-kill curve and effect of EDTA on the antibacterial activity of PlyE146. For the time-kill experiments (A), logarithmic-phase growing *E*. *coli* K12 cells were exposed to PlyE146 (400 μg/mL) in NaPi buffer (pH, 6.0) for 4 h at 37°C. For EDTA experiments (B), logarithmic- and stationary-phase growing *E*. *coli* K12 cells were exposed to PlyE146 (400 μg/mL) in the absence or the presence of EDTA (5 mM) in NaPi buffer (pH, 6.0) for 2 h at 37°C. Results represent the mean ± standard deviation of triplicate experiments.

To get a further insight into the mechanism of action of PlyE146, the antimicrobial activity was monitored over time under the aforementioned conditions in a time-kill experiment. As depicted in Fig 4A, the killing activity of PlyE146 started only after 60 min incubation (2.5 log_10_ CFU/mL decrease of viable *E*. *coli*) and became bactericidal (3.6 log_10_ CFU/mL decrease) at 2 h. No further reduction was observed at 4 h. Based on these findings, we tested whether pre-treatment of *E*. *coli* K12 cells with the outer membrane permeabilizer EDTA could enhance PlyE146 activity, as observed with other lysins [23]. Unexpectedly, addition of 5 mM of EDTA did not have any effect on the activity of PlyE146 against *E*. *coli* (both logarithmic and stationary phase cells) (Fig 4B), suggesting that PlyE146 can effectively penetrate the outer membrane without prior destabilization and therewith reach the peptidoglycan substrate.

### Spectrum of antibacterial activity of PlyE146

**Fig 5:**
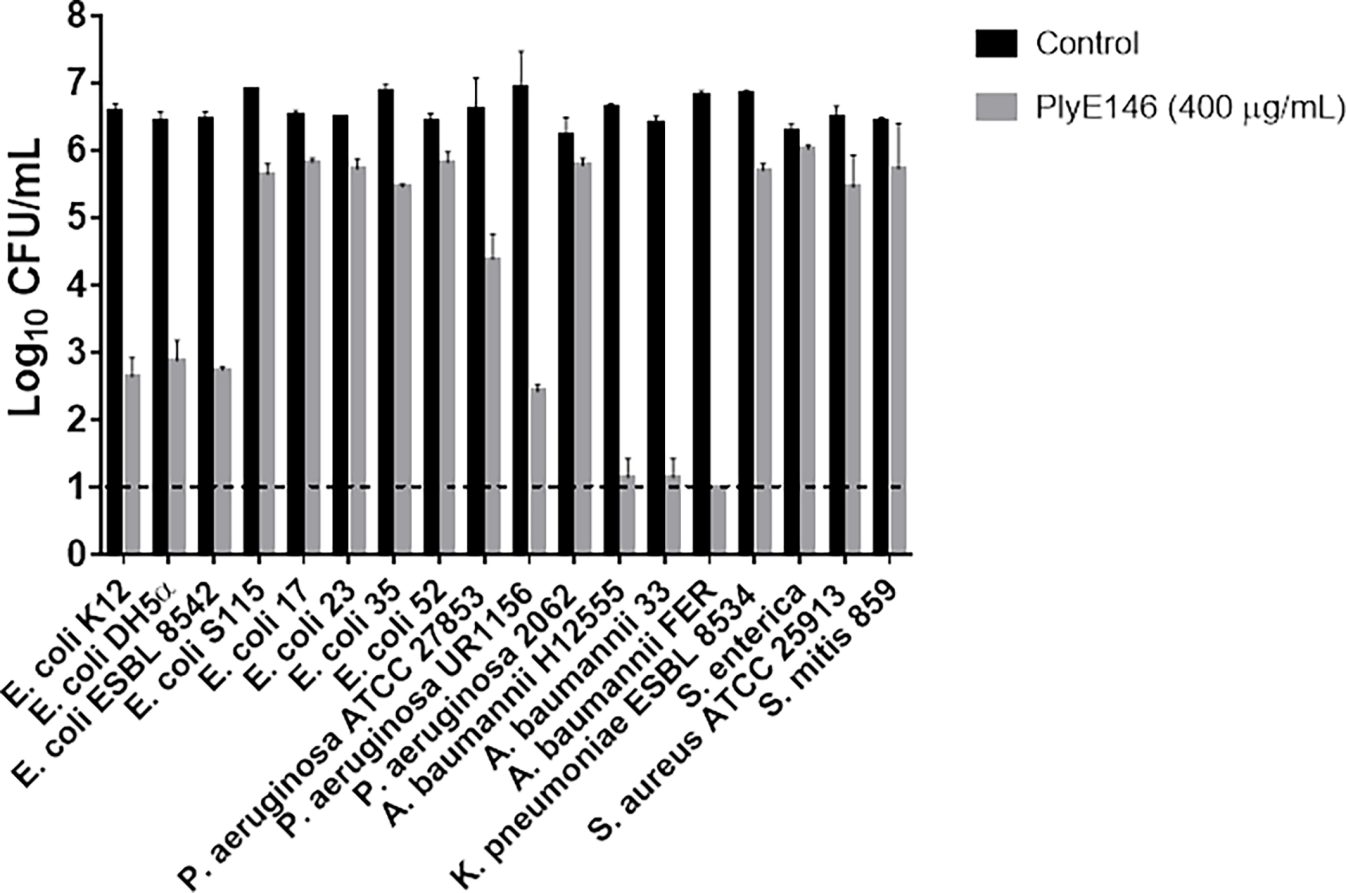
Antibacterial activity of PlyE146 against different Gram-negative and Gram-positive bacteria. The assays were conduct with bacteria in logarithmic growing phase, incubated with 400 μg/mL of PlyE146 in NaPi buffer (pH 6.0) at 37°C for 2 h. The dashed line indicates the limit of detection.

The spectrum of activity of PlyE146 was tested against a panel of 16 Gram-negative bacteria and 2 Gram-positive bacteria. As shown in Fig 5, the antimicrobial activity of PlyE146 was strain specific. PlyE146 efficiently killed 3 out of 8 *E*. *coli* (*E*.*coli* K12, *E*. *coli* DH5α and *E*.*coli* ESBL 8542; >3.5 log_10_ CFU/mL decrease) but was less active against 5 other strains (ca. 1 log_10_ CFU/mL decrease). A similar pattern of activity was observed against *P*. *aeruginosa*; while the activity towards strains ATCC 27853 and UR1156 was bactericidal (3.1 to and 3.9 log_10_ CFU/mL decrease, respectively), strain 2062 was not susceptible to the lysin. A strong activity of PlyE146 was observed against the three tested *A*. *baumannii* strains with ≥4.9 log_10_ CFU/mL decrease. No activity was observed against the tested *K*. *pneumoniae*, *S*. *enterica*, *S*. *aureus* and *S*. *mitis* strains (Fig 5).

### Antibacterial activity of PlyE146 in serum

The activity of PlyE146 was tested in decomplemented human serum against *E*. *coli* K12, *P*. *aeruginosa* UR1156 and *A*. *baumannii* FER strains, three isolates that were efficiently killed *in vitro* (see Fig 5). Interestingly, in the presence of serum, no activity of PlyE146 was recorded against any of the tested bacteria.

### TEM

**Fig 6:**
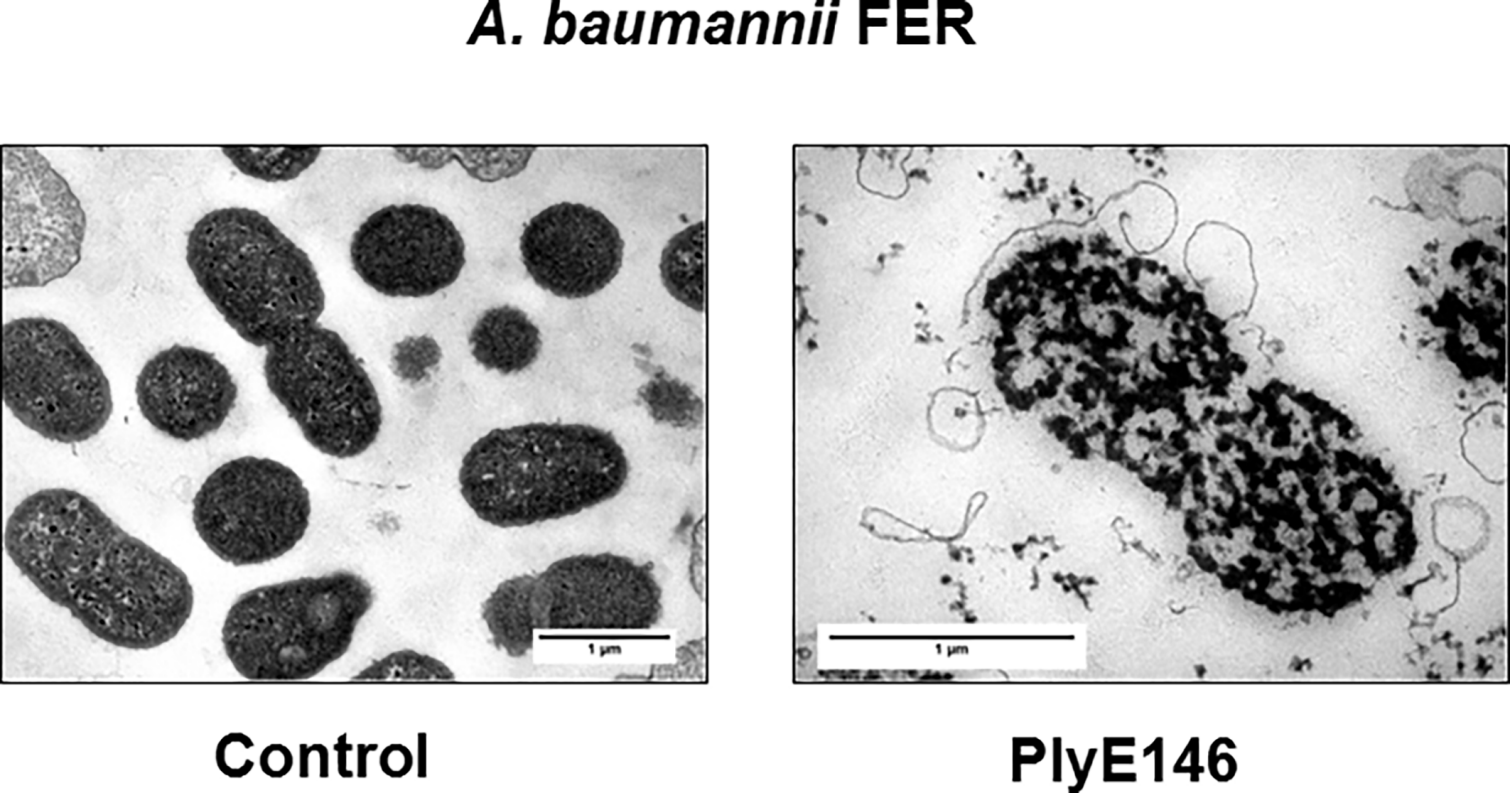
TEM. Representative TEM images of 50 nm sections of untreated control (left panel) and PlyE146-treated (right panel) *A*. *baumannii* (strain FER). TEM images show an evident disruption of the cell wall after exposure to PlyE146 (400 μg/mL) for 1 h.

The effect of the PlyE146 on *A*. *baumannii* structure was visualized by TEM on 50 nm sections. As shown in Fig 6, exposure to 400 μg/mL of PlyE146 led to drastic changes in intracellular density and to the disintegration of the cell wall and the cell membrane.

## Discussion

Gram-negative bacteria are becoming increasingly resistant to antibiotics and this poses a problem for the treatment of the associated infections. Due to the shortage of novel antibiotics, innovative strategies must be developed. Lysins are phage-related enzymes that hydrolyze the bacterial peptidoglycan and therefore represent an attractive option to tackle this issue. However, due to the presence of the outer membrane that hinders the access of the lysins to the peptidoglycan of Gram-negative bacteria, lysins have been for long time considered useful only against Gram-positive bacteria. Notwithstanding, recent works have shown that lysins (natural or engineered) do possess antibacterial activity against *P*. *aeruginosa* and *A*. *baumannii* [8,17,19,20], raising the prospect of a possible use for treating Gram-negative bacterial infections too.

A common feature of lysins active against Gram-negative bacteria is the presence of a highly cationic peptide at the C- or N-terminus that can potentially destabilize the outer membrane of Gram-negative bacteria and give access to the peptidoglycan. This notion has been supported by the work of Lood and others [19], which showed that the highly positively charged C-domain of a phage lysin active against *A*. *baumannii* was sufficient to kill the bacteria. Inspired by these observations, we searched similar lysins with antimicrobial activity towards *E*. *coli* and identified PlyE146, a putative lysin with a predicted highly positively charged C-terminal domain and an N-acetylmuramidase (lysozyme) N-terminal domain. PlyE146 exhibited a significant lethal activity against several *E*. *coli* and *P*. *aeruginosa* strains with a > 3 log_10_ CFU/mL decrease after 2 h of exposure. However, the antimicrobial activity against *E*. *coli* and *P*. *aeruginosa* varied from strain to strain. To our knowledge, this is the first report of an endolysin with significant intrinsic antibacterial activity towards *E*. *coli*. The only endolysins with a reported killing activity against *E*. *coli* are the fusion *E*. *coli* phage Lysep3 lysins, which exhibited a maximal activity comparatively lower than that of PlyE146 (slightly above of 1 log_10_ kill after 10 h exposure) (23). The killing activity of PlyE146 was much greater against *A*. *baumannii* and resulted in ca. 5 log_10_ CFU/mL decrease for three clinical isolates tested. By contrast, no activity was detected against *K*. *pneumoniae* and *S*. *enterica* isolates. This species and strain-specificity, together with the apparent greater sensitivity of *A*. *baumannii*, might be ascribed to different features at the level of the outer membrane structure, like a different composition in the lipopolysaccharides, which may prevent or facilitate the access to exogenous lysins [23]. Consistent to what observed for other endolysins with intrinsic activity towards Gram-negative bacteria [19], PlyE146 exerted its optimal activity at pH 6.0, suggesting a central role played by the cationic peptide in providing access to the peptidoglycan substrate. Indeed, at low pH the cationic peptide becomes more protonated and may thereby destabilize more efficiently the outer membrane of Gram-negative bacteria through the interaction with the negatively charged phosphates of the outer membrane by interfering with the stabilizing divalent cations. Consistent to this notion, pretreatment of cells with the membrane-permeabilizer EDTA [8] did not improve the activity of PlyE146. Of note, the presence of a 6-His tag in the proximity of P146 might have had an effect on the putative translocating activity mediated by P146, a possibility that is for further studies to determine.

In contrast to the fast kinetics generally observed with Gram-positive lysins [6] or the artificial lysin Art-175, where cell explosions could be observed within 5 min of exposure, the effects of PlyE146 on *E*. *coli* K12 cells could be observed only after 30 min exposure and reached a similar killing efficacy after 2 h treatment. This finding, which is in agreement with the results obtained with for PlyF307, further supports the scheme of a two-step mechanism with an initial slow penetration of the outer membrane followed by the hydrolysis of the peptidoglycan [19], as evidenced by the TEM analysis of *A*. *baumannii* cells after 2 h exposure to PlyE146. Remarkably, even in the presence of a clear dispersion of the cell wall, *Acinetobacter* cells do not appear to explode, as it generally occurs with Gram-positive lysins [13] or Art-175 [9], where cells burst after the bulging of the cytoplasmic membrane through punctured holes in the cell wall. Although the exact mode of killing is for further studies to be determined, the slower activity of PlyE146 is likely behind this killing effect.

As expected, the activity of PlyE146 against *E*. *coli* cells in logarithmic phase was much greater as compared to cells in stationary phase. Indeed, lysins have been reported to be generally less active against stationary-phase cells than against logarithmically growing cells [13]. This effect can be ascribed to the thinner peptidoglycan layer at the level of the septum in growing cells, which makes it an easier pray for the lysin [13].

As previously shown for other lysins [21,23], PlyE146 became inactive against *E*. *coli*, *P*. *aeruginosa* and *A*. *baumannii* in serum. Absence of activity in serum as well as under physiologically relevant conditions, like in the presence of salts and at physiological pH 7.4, at the moment precludes a potential use of PlyE146 as a therapeutic agent. A study is undergoing in the laboratory to investigate whether modifications thereof can revert this loss of function.

In conclusion, we characterized a new *E*. *coli* prophage lysin able to efficiently kill different Gram-negative bacteria in vitro. This study further demonstrates that bacteriophage lysins represent a promising alternative to antibiotics in the fight against MDR Gram-negative bacterial pathogens.
